# Genital involvement and psoriasis severity

**DOI:** 10.1016/j.jdin.2026.02.005

**Published:** 2026-02-24

**Authors:** Vitoria Regina Pedreira de Almeida Rêgo, Alice Rocha de Magalhães, Maria de Fátima Santos Paim de Oliveira, Fabíola Leal Silva, Fernanda Pedreira Rêgo Rocha, Juliana Dumet Fernandes

**Affiliations:** a Dermatology Department, Medical School of Bahia, Federal University of Bahia (FAMEB/UFBa), Salvador, BA, Brazil; b Dermatology Division, Prof. Edgard Santos School Hospital Complex, Federal University of Bahia (C-HUPES/UFBa), Salvador, BA, Brazil; c Clinical Immunology and Allergy Division, University of Sao Paulo, Sao Paulo, Brazil

**Keywords:** epidemiology, genital diseases, psoriasis, quality of life

**Figure:**
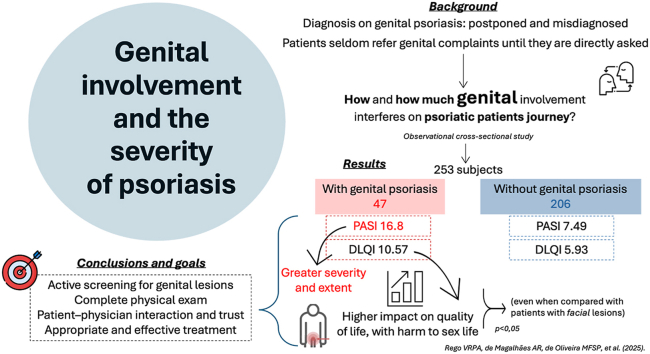

*To the Editor:* Genital psoriasis is a frequent yet often underdiagnosed manifestation of psoriasis, with significant psychosocial and clinical implications. Although up to 63% of patients develop genital lesions during their disease course, many do not report these symptoms unless specifically questioned, resulting in delayed diagnosis and undertreatment.^1^^,^^2^

We conducted a cross-sectional study at the Psoriasis Clinic of the Dermatology Service, Federal University of Bahia (Brazil), including 253 adults followed for 5 years. Participants were classified according to genital involvement: 47 (18.6%) had genital lesions, while 206 (81.4%) did not. For further analysis, patients with facial lesions were also separated. Disease severity and quality of life were assessed using the Psoriasis Area and Severity Index (PASI) and the Dermatology Life Quality Index (DLQI), compared by their values and by categories: mild and moderate/severe (Table I). Continuous variables were summarized as mean, and categorical variables as proportions. Normality was assessed using the Kolmogorov–Smirnov test. Categorical variables were compared using the chi-square or Fisher’s exact test, as appropriate. Comparisons between 2 groups were performed using Student’s *t* test for normally distributed variables and the Mann–Whitney *U* test otherwise. Kruskal-Wallis test was applied for comparison among 3 groups. All tests were 2-tailed, and *P* < .05 was considered statistically significant. The mean age of participants was 42.5 years, and 51.4% were male. The most common clinical forms were vulgar (67.1%) and arthropathic psoriasis (20.5%). Patients with genital involvement presented significantly higher PASI and DLQI scores compared with those without genital lesions (*P* < .05) (Table I). Mean PASI values were 17.0 for patients with genital lesions (with or without facial involvement), 10.1 for those with facial but nongenital lesions, and 4.5 for those without facial or genital involvement. Mean DLQI scores followed the same pattern: 10.5, 6.9 and 5.3, respectively (Table II). A greater proportion of patients with genital psoriasis had undergone 3 or more previous treatment modalities, suggesting a more severe and refractory disease course.

**Table I:** Demographic data and severity classification comparing groups with genital psoriasis and without genital psoriasis

	Psoriasis genital involvement	
	Yes	No
Total = 253	47 (18.6%)	206 (81.4%)
Sex		
Female	25 (53.2%)	81 (39.3%)
Male	22 (46.8%)	125 (60.7%)
Clinical presentation		
Vulgaris	41 (87.2%)	191 (92.7%)
Erythrodermic	3 (6.4%)	6 (2.91%)
Guttate	2 (4.3%)	2 (0.97%)
Pustular	1 (2.1%)	7 (3.39%)
Age (mean - years)	45.91	52.71
Age at diagnosis (mean - years)	34	38.45
Number of previous treatments		
>3	15∗	20∗
<3	32∗	186∗
PASI		
Median	16.8†	7.49†
<10 (mild)	19	93
>/= 10 (moderate/severe)	28	113
DLQI		
Median	10.57†	5.93†
<5	13 (28.3%)	102 (49.5%)
>/= 5 (moderate/severe)	34 (71.7%)	104 (50.48%)

*DLQI*, Dermatology Life Quality Index; *PASI*, Psoriasis Area and Severity Index.

∗ A statistically significant difference between groups was observed using chi-square test (*P* < .05).

† Mann–Whitney *U* test showed that PASI scores were higher in patients with genital involvement (median, 16.8) than in those without genital lesions (median, 7.49) (*U* = 2192.0; *P* < .01); DLQI scores were also higher, with statistical significance (*P* < .01).

**Table II:** Association between Psoriasis Area and Severity Index, Dermatology Life Quality Index, and presence of genital lesion

Metrics	Psoriasis involvement		
	Facial and genital lesions	Facial with NO genital lesions	Absence of facial and genital lesions
PASI (mean)	17.02∗	10.08	4.54
DLQI (mean)	10.54†	6.98	5.31

*DLQI*, Dermatology Life Quality Index; *PASI*, Psoriasis Area and Severity Index.

∗ Comparisons among groups were performed using the Kruskal–Wallis test, with a 2-tailed *P* < .05 considered statistically significant. PASI scores were significantly higher in patients with genital lesions compared with the other groups.

† DLQI scores were also higher in this group (*P* < .05).

Genital involvement was associated with a markedly impaired quality of life, consistent with prior studies.^3^, ^4^, ^5^ The burden extends beyond sexual activity, affecting self-esteem, interpersonal relationships, and emotional well-being. Nevertheless, genital symptoms remain underdiscussed due to embarrassment and insufficient inquiry during dermatologic visits. Active screening and patient education are crucial for proper diagnosis and management.^2^

This study is limited by its single center design and the absence of genital specific assessment tools, such as the Static Physician`s Global Assessment of Genitalia and the Genital Psoriasis Sexual Frequency Questionnaire.^4^^,^^5^ However, our findings demonstrate that widely available instruments like PASI and DLQI can effectively capture clinical and psychosocial impact of genital psoriasis.

In conclusion, genital psoriasis is associated with greater disease severity and reduced quality of life. Routine evaluation of genital areas in patients with psoriasis should be incorporated into dermatologic practice to ensure comprehensive and empathetic care. As genital psoriasis may be correlated with extensive disease, treatment refractoriness, and psoriatic arthritis, defining its severity is challenging and subject to confounding factors.

## Conflicts of interest

None disclosed.

## References

[1] Boehncke W.H., Schön M.P. (2015). Psoriasis. Lancet.

[2] Meeuwis K.A., de Hullu J.A., Massuger L.F.A.G., van de Kerkhof P.C.M., van Rossum M.M. (2011). Genital psoriasis: a systematic literature review on this hidden skin disease. Acta Derm Venereol.

[3] Ryan C., Sadlier M., De Vol E. (2015). Genital psoriasis is associated with significant impairment in quality of life and sexual functioning. J Am Acad Dermatol.

[4] Strober B., Ryan C., van de Kerkhof P. (2020). Recategorization of psoriasis severity: Delphi consensus from the International Psoriasis Council. J Am Acad Dermatol.

[5] Cather J.C., Ryan C., Meeuwis K. (2017). Patients' perspectives on the impact of genital psoriasis: a qualitative study. Dermatol Ther (Heidelb).

